# MeCP2 and the enigmatic organization of brain chromatin. Implications for depression and cocaine addiction

**DOI:** 10.1186/s13148-016-0214-5

**Published:** 2016-05-21

**Authors:** Juan Ausió

**Affiliations:** Department of Biochemistry and Microbiology, University of Victoria, Victoria, BC V8W 3P6 Canada

## Abstract

Methyl CpG binding protein 2 (MeCP2) is a highly abundant chromosomal protein within the brain. It is hence not surprising that perturbations in its genome-wide distribution, and at particular loci within this tissue, can result in widespread neurological disorders that transcend the early implications of this protein in Rett syndrome (RTT). Yet, the details of its role and involvement in chromatin organization are still poorly understood. This paper focuses on what is known to date about all of this with special emphasis on the relation to different epigenetic modifications (DNA methylation, histone acetylation/ubiquitination, MeCP2 phosphorylation and miRNA). We showcase all of the above in two particular important neurological functional alterations in the brain: depression (major depressive disorder [MDD]) and cocaine addiction, both of which affect the MeCP2 homeostasis and result in significant changes in the overall levels of these epigenetic marks.

*“Au temps déjà lointain où, étudiant de la sublime Science, nous nous penchions sur le mystère tout rempli de lourdes énigmes…”*

Fulcanelli (1925), Le mystère des cathédrales [1]

## Background

It was not until 1999, when it was realized that mutations in MeCP2 may result in RTT (an autistic type of neurodevelopmental disorder) [2], that the scientific community began to pay a great deal of attention to this protein. A significantly large amount of research has been carried out since that time, and what was initially thought to be a relatively simple, unique repressor protein [3], developed into a fascinating transcriptional regulator [4, 5]. Part of its functional multiplicity is due to the intrinsically disordered conformation of MeCP2 [4, 6], which makes it amenable to interaction with multiple interacting protein partners, as well as to its labelling with various post-translational modifications (PTMs), such as phosphorylation, acetylation and ubiquitination [4]. Such PTMs further modulate the interaction of MeCP2 with chromatin [7].

In the brain, where MeCP2 is highly abundant [8, 9], it can bind to both DNA methylated and non-methylated regions of chromatin [10]. Yet, the chromatin organization resulting from these interactions is still unclear, and the molecular details involved are not completely understood. The large occurrence of MeCP2 in neurons has led to the realization in recent years that alterations in the homeostatic levels of the protein can have important functional consequences for several neurodevelopmental and neurodegenerative diseases that highly transcend RTT [4]. In this regard, a significantly large amount of research and information has been recently gathered about two types of brain alterations, which have important societal implications: depression [11] and cocaine addiction [12]. Both have a strong epigenetic component that involves changes in MeCP2 levels and alterations of the histone PTMs (such as acetylation and phosphorylation). The molecular details regarding the involvement of MeCP2 in these brain disorders has brought about a significant amount of exciting functional information, relevant not only for the particular mechanisms involved in each of them, but more importantly, for the overall molecular biology of MeCP2 in the brain.

## A brief history of MeCP2: Cancer, Rett syndrome, MDD and cocaine addiction

In 1989, the search for a protein “reader” that specifically binds to regions of methylated CpG in the mammalian genome [13] led to the identification of a protein known as MeCP-1 [14], a protein that binds *in vitro* to DNA sequences containing at least 12 symmetrically methylated CpGs [15]. Three years later, an additional methylated CpG binding protein was identified called MeCP2 [16, 17]. Due to its 5-methyl cytosine (5mC) binding activity, the protein was initially assigned a repressive role, supposedly acting at the methylated regions of transcriptionally repressed genes [3]. However, more recently, the protein has been shown to additionally bind to 5-hydroxy-methylated cytosines (5hmC) at the elongation regions of transcriptionally active genes [18]. It has also been shown to bind to both repressive and activating co-factors [19, 20]. Therefore, MeCP2 should be viewed as a transcriptional regulator [4]. By virtue of its ability to bind to methylated DNA and to chromatin modifying complexes, such as HDACs and CREB, MeCP2 can be considered both an epigenetic ‘reader’ and a ‘writer’.

The initially identified form of MeCP2 turned out to be what is today known as the MeCP2-E2 isoform. It was not until 2004 that the MeCP2-E1 isoform (initially called MeCP2 B) was discovered [21]. MeCP2-E1 is the major form of MeCP2 [22] and the one that is most abundant in the brain, corresponding to approximately 90 % of the total MeCP2 in this tissue [23]. Of note, the predicted half-life of the two isoforms is very different, with that of the E1 isoform (4 hours) being significantly different from that of E2 (100 hours) [4].

**Fig. 1:**
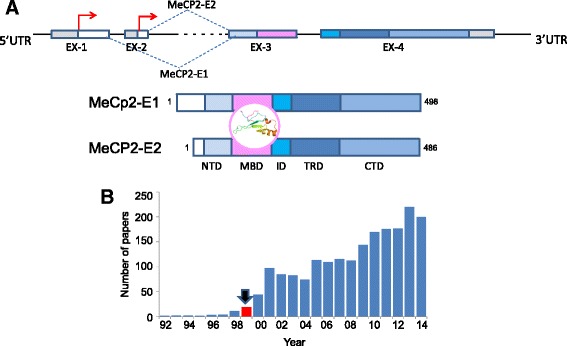
**a** Gene organization of MeCP2. Two isoforms E1 and E2 result from alternative splicing. The red arrows indicate the starting sites of transcription. The domain organization of the resulting protein isoforms is also shown. CTD; C-terminal domain; ID: intervening domain; MBD: methyl-binding domain; NTD; N-terminal domain; TRD: transcriptional repression domain. **b** Number of publications since MeCP2 was first described

The gene organization and schematic representation of the protein structure of these two MeCP2 isoforms are shown in Fig. 1a. The MeCP2 gene is negatively regulated by the high mobility group nucleosome binding (HMGN) of non-histone chromosomal proteins [24], and positively regulated by the transcription factor myocyte enhancer factor 2C (MEF2C) [25]. MicroRNAs, such as miR-132 [26], miR-7b development [27], miR-483-5p [28], miR-155/miR-802 [29] and miR-181 [30] also play a very important role in the regulation of the expression of the gene.

At the protein level, the common methyl binding domain (MBD) (Fig. 1a) is the only region of these proteins that shows a well-characterized tertiary structure [31] within what it is one of the best examples of an intrinsically disordered protein [4]. As such, the protein is subject to numerous PTMs [4], of which the phosphorylation of serine residues at position 80 (S80 of human E2 isoform) [32] and 421 (S421of mouse E1 isoform) [33], commonly referred to as S80 and S421 [34], have been the most extensively characterized. Importantly, the levels of MeCP2 and its homeostasis in the brain (where the protein is more abundant) appear to be critical to its proper developmental function [35], although normal physiologically-relevant fluctuations can occur in a circadian-regulated manner [36].

It took seven years since the discovery of MeCP2 for the number of publications on this protein (Fig. 1b) and the general interest of the scientific community to pick up on Huda Zoghbi’s team demonstration at the Baylor College of medicine, which demonstrated that RTT, for the most part, is due to mutations in MeCP2 [2]. Rett syndrome is an X-chromosome-linked neurodevelopmental disease predominantly affecting girls. It is related to autism and is characterized by intellectual disability and developmental regression [37]. Studies published previous to the knowledge of the connection of MeCP2 with RTT included the characterization of the interaction of MeCP2 with histone de-acetylases (HDACs) [38]. Together with the interaction of MeCP2 with CpG methylated DNA, this finding reinforced the notion of a transcriptionally repressive role initially associated with this protein [39], while establishing the first link between MeCP2 and histone acetylation. This concept fits well with that of DNA CpG hypermethylation, which is usually found at the promoters of tumor-suppressor genes in many cancers [40–43] where MBD proteins, including MeCP2, play an important role [44]. A paper appeared during the interim period of time that preceded the RTT-MeCP2 association, which involved MeCP2 in the repression of the retinoblastoma gene [45], and it paved the way in terms of MeCP2’s involvement in cancer. Indeed, MeCP2 has been recently recognized as playing the role of a bona fide oncogene [46].

**Fig. 2:**
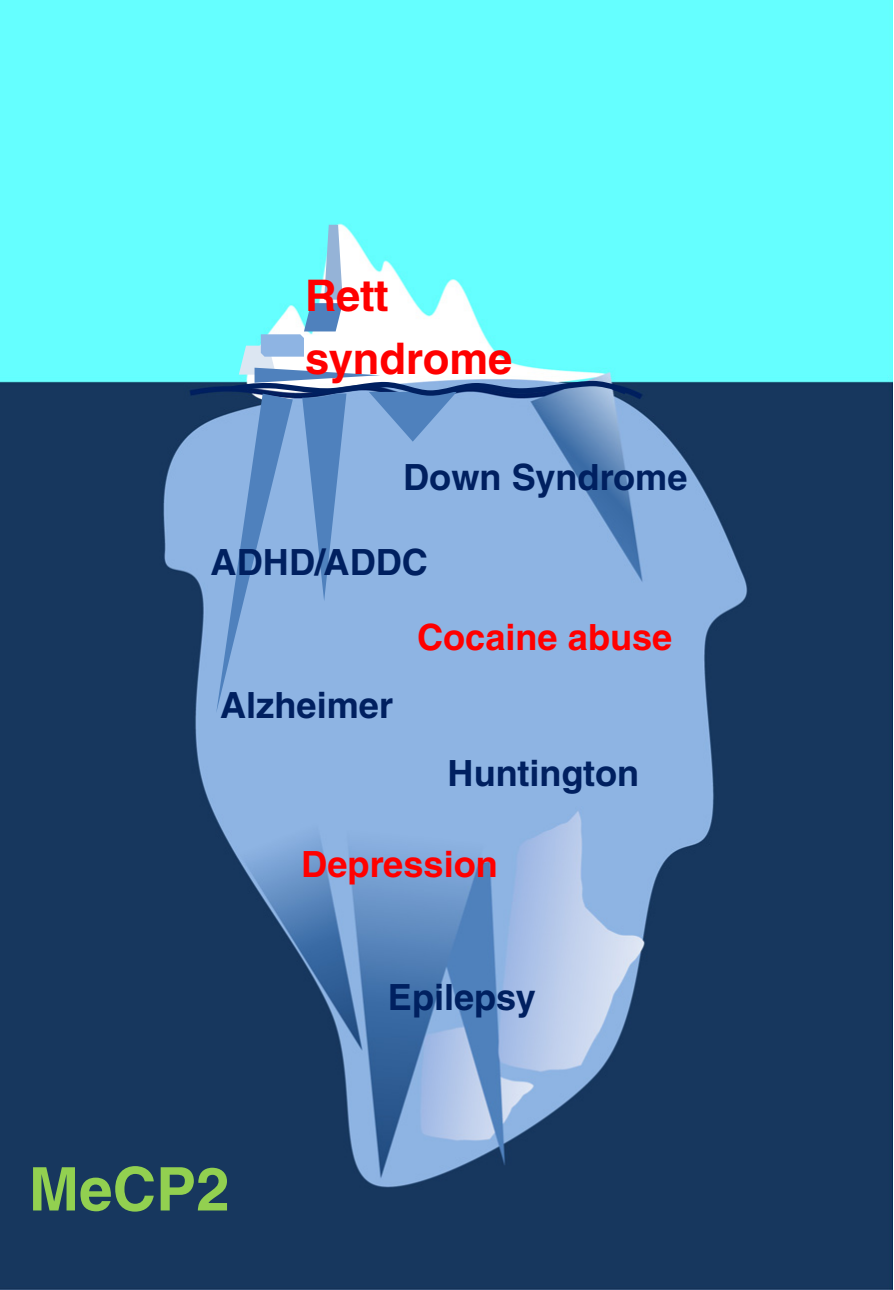
The involvement of MeCP2 in Rett syndrome neurodevelopmental disease represents just the tip of the iceberg. The high abundance of MeCP2 in the brain [8, 9] has implications for many other neuropathological disorders [4]

Although the presence of MeCP2 in the brain is exceedingly larger than in any other tissue [9], the last sentence of the prior paragraph should serve as a reminder that the protein plays additional important roles in many other tissues and physiological aspects of the body. An underscoring example of this can be drawn from a recent observation describing a decrease in both the levels of mRNA and of MeCP2 itself during chronic heart failure [47]. The same holds equally true for the brain and, in this case, it is the massive presence of MeCP2 in this organ that is responsible for many other neurodevelopmental and neurodegenerative processes (Fig. 2) [4] far beyond RTT. As will be discussed later, alterations in the levels of MeCP2 have been described, for both major depressive disorder (MDD) and cocaine addiction [48], two brain affections which pervade our modern society and are an important public health concern [49–51].

## Chromatin, the variance of the histones and chromatin epigenetics

Chromatin is the name given to the nucleoprotein complex that results from the association of DNA and histones. Histones are basic proteins, rich in both Lysine and Arginine residues. They have been broadly classified into two structurally different major groups [52]: core and linker histones. Core histones (H2A, H2B, H3 and H4) form an octameric protein complex consisting of 2 H2A-H2B dimers and an H3-H4 tetramer that serves as a protein core, around which 145-147 bp DNA is wrapped in approximately one and three quarters of left-handed super-helical turns to form the nucleosome core particle (NCP). In the chromatin fiber, NCPs are connected to each other by shorter linker DNA regions of variable nucleotide length. The average distance in nucleotides between the centers of two adjacent nucleosomes is known as the nucleosome repeat length (NRL), which may vary between different tissues, with neurons exhibiting an unusually short one (160 bp) in metazoan tissues [53]. Linker histones (of the histone H1 family) bind to the NCP close to its dyad axis, at the entry and exit sites of the linker DNA connecting adjacent nucleosomes [52, 54]. The former have been further classified into canonical replication-dependent histones and replication-independent replacement histone variants (*i.e.* H2A.Z, H2A.X, H2A.B, macro H2A, H3.3) [55].

However, what we have over the years referred to as histone variants [56] due to their lower abundance in metazoans may have been the primordial canonical histones, while what we are currently calling replication-dependent canonical histones may actually represent the real variants. As a matter of fact, most protein-encoding eukaryotic genes, in contrast to the replication-dependent histone genes, contain introns and are poly-adenylated, as compared to the replication histone genes, which are intron-less and are poly A- [57]. Of note, yeast H2A is H2A.X and H3 is H3.3 [58]. Moreover, H2A.X in metazoans is encoded by both poly A+ and poly A- mRNAs [59]. It appears that as genomes became increasingly larger, there was a need for specialization in the histone genes to adapt to the synthesis demands that ensure the fast and efficient coverage of the genomic DNA during DNA replication. This was achieved by a completely different regulation of the expression of these genes, as well as the loss of their introns and poly-adenylation [57]. Indeed, some of these replication-dependent histones are actively expressed in differentiating and aging retinal neurons at a time when replication has already ceased [60].

Regardless of their ancestry or origin, replication-independent histone variants play an important role in brain development [61, 62]. A few examples have been recently brought to the forefront. Histone H2A.Z has been shown to play a critical role in recent and remote memory consolidation [63], and histone H3.3 has been shown to play an essential role in neuronal plasticity and cognition [64].

In addition to the histone variation, histones in general, regardless of their replication dependent or independent nature, can be post-translationally modified (PTM) at specific amino acid sites in their sequence. These chemical modifications (*i.e.* acetylation, methylation, phosphorylation and ribosylation, to name just a few [65]) set the molecular basis for the proposal of the histone code fifteen years ago [66]. From then on, chromatin went from being considered a passive structural template that sustained the DNA metabolic functions to acquiring an epigenetic role of its own, in which the histones play a crucial role. As a matter of fact, histone variants and histone PTMs provide the molecular basis for their epigenetic involvement [55, 67, 68].

The histone code involves a complex network of protein ‘writers’, ‘erasers’ and ‘readers’ responsible for the downstream functional implications of the histone PTMs. However, not all histone variants and PTMs have an exclusive epigenetic function. For instance, in addition to its epigenetic involvement, global histone acetylation of pan-acetylated H3/H4/H2A.Z can alter the structural organization of chromatin on their own [69], as is also the case in H4 K16 acetylation [70].

In addition to histone variants and their PTMs [71], other chromatin trans-acting factors, such as MeCP2, by virtue of binding to methylated [72] and hydroxymethylated [18] DNA could also have an important involvement, thus connecting epigenetics and neuronal function [73]. How MeCP2 and histone epigenetic baggage affect neuronal chromatin organization is still one of the many remaining enigmas that will be analyzed in the next two sections.

## MeCP2 and the chromatin organization of neurons

As we and others have experimentally shown, MeCP2 is a very abundant chromosomal protein within the brain, with approximately one MeCP2 mole for every nucleosome in the neurons of an adult brain [8, 9]. The question then arises as to how this high MeCP2 prevalence is accommodated within the histone crowded chromatin ensemble. Moreover, the levels of MeCP2 in neurons increase during brain development, and are accompanied by a decrease in the NRL from approximately 200 bp at the early embryonic stages to 160 bp in the adult brain [74]. In addition, it has long been known, and recently corroborated, that the levels of linker histones (histones of the H1 family) in the brain are approximately 50 % of what would be present in other somatic tissues [8, 75]. It was initially shown that MeCP2 is able to displace histone H1 in vitro in a DNA methylation-dependent manner [76]. When all this information is considered together, the question arises as to whether MeCP2 is present in a quasi-even alternating nucleosome arrangement, or clustered in regions that are highly enriched, others in which H1 prevails, or a mixture of the two.

**Fig. 3:**
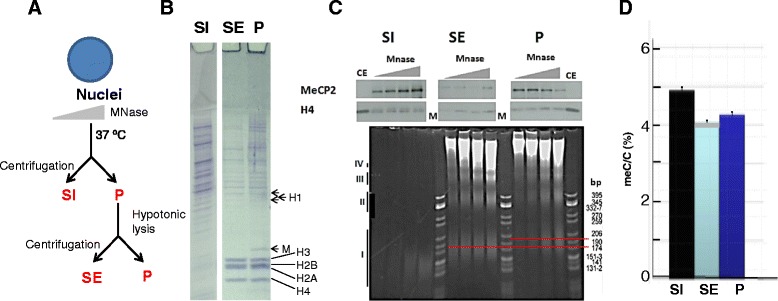
**a** After micrococcal nuclease (MNase) digestion of cellular nuclei, small-sized nucleosomes chromatin (nI) (see Fig. 4) that leak through the nuclear membrane pores can be recovered in the supernatant (SI) after centrifugation. The nuclear pellet can next be hypotonically lysed in the presence of 0.25 mM EDTA and centrifuged once more to yield a supernatant (SE) fraction and an insoluble pellet (P). **b** Protein composition of the SI, SE and P fractions as analysed by polyacrylamide gel electrophoresis (PAGE) in the presence of SDS detergent. Histones H1, H2A, H2B, H3 and H4 are indicated, as well as myelin (M). **c** Analysis of the SI, SE and P fractions during a time-course Mnase digestion of rat whole brain nuclei. The upper part of the Figure shows a Western blot analysis using MeCP2 and H4 antibodies. The lower part shows a native PAGE analysis of the DNA composition of the fractions obtained at different time of digestion. CE: chicken erythrocyte histones used as a control; M: pBR322-*Cfo* I–digested DNA used as a marker. The numbers on the right had side of the native PAGE indicate the DNA fragment sizes in base pairs (bp). The red lines highlight the shift in the center of the mononucleosome DNA (nI) distribution in SE and P. **d** Relative meC/C percentile composition of the SI, Se and P fractions at limit Mnase digestion. (Section **c** was reproduced from Fig. 2A from [9], with permission)

With regards to the potential role of MeCP2 in the organization of chromatin, and neuronal chromatin in particular, one of the most puzzling and yet unexplained observations is that of its early release during micrococcal nuclease digestion [9]. Using a relatively simple chromatin fractionation method (Fig. 3a), it was demonstrated that a large amount of MeCP2—more than 50 percent—is released into a supernatant S1, which is deficient in histones (particularly H1) (Fig. 3b) and which consists of mono-nucleosomes (Fig 3c) along with small oligonucleotides that are released during nuclease digestion and contain a higher level of methylated cytosine (Fig. 3d) [9]. Thus, a significant amount of MeCP2 appears to be bound to chromatin regions that are highly accessible to micrococcal nuclease. Such an observation is highly enigmatic and appears to be in contrast with the initial repressive role associated with the protein [3], as well as with the highly compacted nucleoprotein complexes it forms in vitro upon interaction with nuclesosome arrays [77]. Interestingly, as shown in Fig. 3c, during the early stages of digestion, most of the MeCP2 present in S1 appears to arise from the highly compact “insoluble” structures present in fraction P.

Equally intriguing is the difference in NRL observed between chromatin in the P fraction (approx. 200 bp) and that of the SE fraction (centered at approx. 174 bp). This difference is reminiscent of the transition in NRL observed during neuronal development (from 200 bp at the onset of development to 160-170 bp in mature neurons [74]) in a way that is independent of the chromatin H1 content [53]) and for which there is yet no explanation. Interestingly, the NRL change is dependent on developmental factors of the brain, such as thyroid hormone [78], and follows the transition of the neuronal nucleus from a small heterochromatic to a larger euchromatic nucleus [79, 80]. Whether all these transitions are dependent on MeCP2 remains to be determined.

**Fig. 4:**
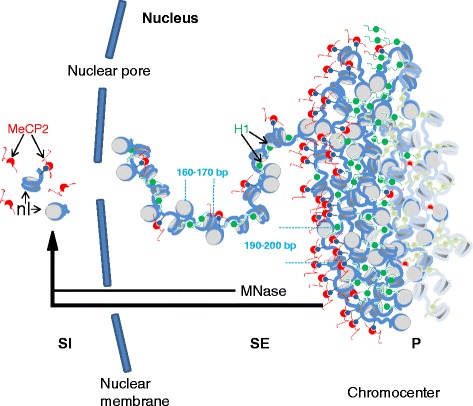
Hypothetical model for the MeCP2 distribution within the neuronal chromatin organization. MeCP2 is sparsely distributed in the chromatin fibers in the nucleoplasm binding to DNA methylated sites that are depleted of H1. An important fraction of MeCP2 binds preferentially to the periphery of chromocenters/nucleoli. Upon micrococcal nuclease (MNase) digestion, MeCP2 is very quickly released from the insoluble pellet-able (P) chromocenter periphery and leaks through the nuclear membrane pores into the SI fraction (see Fig. 2). MeCP2 associated to nucleosomes is also released from nuclear chromatin fibers (SE fraction), albeit at a much lower level as indicated by the thickness of the *arrows*. nl: mononucleosome

Based on these results, we would like to put forward a hypothetical model (Fig. 4) that still requires further experimental testing but which is different than that we had proposed earlier [9]. In this model, an important amount of MeCP2 is concentrated at the chromocenters and the nucleolus periphery, and therefore, while attached to these ‘insoluble’ chromatin domains, it should be highly accessible to digestion by nucleases. Indeed, immuno-gold labelling using electron microscopy initially demonstrated a preferential localization of MeCP2 at the periphery of highly dense chromatin structures [9]. This is in agreement with earlier observations of its co-localisation with DAPI-positive, heterochromatic regions that surround the nucleolus [81]. Of note, most of the transcriptional dynamics in neurons occur in the nucleolus and at different euchromatin sites [82]. Moreover, in neurons, MeCP2 has been shown to bind to the methylated CA sites of exceptionally long genes and, more importantly, disruption of MeCP2 alters the levels of rRNA [72].

The presence of two MeCP2 isoforms with putative, disparately different half-time lives may impinge in the dynamics of neuronal chromatin, giving it a very fluid organization. Although highly speculative, one could envision an organization in which MeCP2 that already binds highly dynamically to chromatin as determined by FRAP [83, 84] could utilize its E1 and E2 isoforms to differentially alter its association with different chromatin domains in a quick response to rapid changes in the environment (such as in response to the circadian cycle [36], or in the short-term response to drugs, such as cocaine [85]). It is likely that the PEST sequences common to both isoforms play an additional important role in such turnover [86]. PEST sequences consist of at least 12 amino acids residues in length and typically signal the protein containing them for rapid proteolytic degradation by the 26S ubiquitin proteasome system (UPS) through phosphorylated serine-mediated ubiquitination at a contiguous lysine residue within the PEST domain [87, 88]. In this regard, although experimental evidence involving these domains is still missing, a ubiquitin ligase (RNF4) [89] has recently been identified that may be involved in the process, and serine 80 and lysine 82 (nomenclature referred to the E2 isoform) within the PEST 1 sequence in mice have been shown to be phosphorylated [32] and ubiquitinated [90] respectively.

The regulation of MeCP2 homeostasis and additional brain epigenetic markers, to be summarized in the following section, provide an extra layer of complexity to this chromatin organization and ultimately are responsible for the chromatin alterations that affect its function in the brain.

## Critical brain epigenetic markers

Four main epigenetic contributors deserve special attention when it comes to the physiologically relevant aspects of MeCP2 and neuronal chromatin in normal and altered functional states of the brain: DNA methylation, MeCP2 phosphorylation, histone acetylation and microRNAs.

### DNA methylation

MeCP2 is a methylated cytosine ‘reader’ and any changes in DNA methylation are going to have immediate downstream effects on the MeCP2-dependent organization of chromatin. Contrary to what was earlier believed, DNA methylation in neurons is highly dynamic and may change quite rapidly to alter the MeCP2 distribution and their connections or synapses. The process involves a group of enzymes called Tet (ten eleven translocation), which initially oxidize 5mC to 5hmC [91] and subsequently lead to active DNA de-methylation [92] in conjunction with the base-excision repair (BER) pathway. Tet 3 has recently been shown to regulate synaptic transmission and homeostatic plasticity through this mechanism [93]. DNA methylation is then restored by Dnmt1 and Dnmt3a DNA methyl-transferases [94] that close the cycle, and thus contribute to the process of synapsis function. Interestingly, as was mentioned previously, MeCP2 binds very tightly to 5hmC [72], and in doing so it plays a very important role in active transcription [18] within mature neurons. Furthermore, the histone variant H2A.X, which increases during neuronal development [61], has been shown to be associated with BER [95, 96] and was the only histone variant found to co-immuno precipitate with MeP2-containing nucleosomes [9].

### MeCP2 phosphorylation

MeCP2 phosphorylation is by far the most studied PTM of this protein. MeCP2 has been reported to be phosphorylated at various serine residues, such as S13, S80, S149, S164, S229, S274, S401 and S421 [32, 90].

Among these MeCP2 serine residues, phosphorylation at residues S80 and S421 have been proposed to have opposite effects on neuronal activity [32, 34, 97]. In neurons at rest, MeCP2 is tightly associated with *Bdnf* promoter III, acting as a repressor. Upon neuron depolarization, and following calcium influx, MeCP2 S421 becomes phosphorylated by a Camk2/4-dependent mechanism [33] and is released from the *Bdnf* promoter III, allowing for its activity-dependent transcription. Phosphorylation at S421 has also been shown to be associated with accurate synapse development and behaviour [33]. In an opposite mode, MeCP2 S80 becomes dephosphorylated upon neuronal depolarization, allowing for the dissociation of MeCP2 from chromatin. Conversely, MeCP2 is phosphorylated at S80 by homeodomain-interacting protein kinase 2 (HIPK2) and contributes to induction of apoptosis [98]. This opposite regulation of MeCP2 by neuronal activity suggests that S421 phosphorylation plays an important role in active neurons, while S80p is more important in resting neurons.

More recently, neuronal depolarization has been shown to result in the phosphorylation of MeCP2 at threonine 308 in the transcriptional repressor domain (TRD) of the protein. Phosphorylation of this residue blocks the interaction of MeCP2 with the nuclear receptor co-repressor (NCoR) complex. Among other effects, this elicits *Npas4* transcription (a transcription factor that promotes the development of inhibitory synapses on excitatory neurons) and the ensuing activation of *Bdnf* transcription [99].

### Histone acetylation

The direct or indirect molecular mechanisms behind the relation between MeCP2 and histone acetylation are obscure and require further analysis. Knockout mice in which MeCP2 expression has been completely ablated exhibit an almost 3-fold increase in the histone acetylation in neurons when compared to the wild type [8]. Conversely, treatment of HeLa cells with sodium butyrate (an inhibitor of histone de-acetylases) decreases the levels of MeCP2 by almost 3-fold (Thambirajah and Ng, unpublished results). While it can be argued that the former is the result of the well-documented interaction of MeCP2 with repressive chromatin remodelling complexes containing HDACs, the latter has no straightforward explanation.

Global histone pan-acetylation results in a highly labile nucleosome [100] and affects the folding of the chromatin fiber in the absence of linker histones [101]. It also decreases the inter-fiber chromatin interactions [102]. MeCP2 has been shown to interact with chromatin in a way reminiscent of the interaction between linker histones and chromatin [4]. Indeed, MeCP2 was shown early on to compete with histone H1 in a DNA-methylation-dependent way [76]. Although treatment of cells with sodium butyrate does not significantly affect the levels of histone H1 [102], histone acetylation has been known to impair the binding of histone H1 to the nucleosome [103]. However, a role of histone acetylation in facilitating MeCP2 binding could not be demonstrated [104].

### MicroRNAs

MicroRNAs represent one of the most recently identified epigenetic constituents. MicroRNAs (miRNAs) are non-coding RNA transcripts that control gene expression by binding to complementary sequences (miRNA response elements; MRE) in the 3'-UTR of target mRNAs, thus controlling their degradation and translation [105]. Furthermore, DNA methylation has been found to be crucial in miRNA biogenesis [106], and MeCP2 long 3'UTR contains conserved MREs for several miRNAs that modulate its expression [107].

As was mentioned earlier, MeCP2 homeostasis plays an important role in the proper functional outcome of this protein. MicroRNA miR-132 has been found to play a crucial role in this regard. Blocking miR-132 in cultured rat neurons results in an increase of MeCP2 expression. This, in turn, increases the expression of *Bdnf* that induces miR-132 and represses MeCP2 translation. Taken together, these findings suggest a feedback loop involved in MeCP2 homeostasis [26].

Another regulatory microRNA, miR-7b, is expressed in various regions of the adult mouse brain, and also targets MeCP2 at the 3'-UTR. Two CpG islands have been identified at the 5'-flanking region of the gene encoding for miR-7b. An increase in the methylation of these CpG islands during postnatal neuron maturation increases the recruitment of MeCP2 to these regions. This inhibits miR-7b gene expression and its repressive effect on MeCP2. Overall, miR-7b acts as a negative regulator of MeCP2 gene expression and is at the same time a down-stream target of MeCP2, forming a bi-directional feedback that may also be important for MeCP2 homeostasis during brain development [27].

In humans, the intragenic miR-483-5p, derived from the gene *Igf2* (insulin-like growth factor – 2), has been found to regulate MeCP2 levels. This miRNA binds to the MREs in MeCP2 3'-UTR and inhibits MeCP2 gene expression. miR-483-5p is enriched in the fetal brain and is down-regulated after birth, thus controlling MeCP2 expression during human brain development [28].

The miR-155 and miR-802 also target MeCP2 and have recently shown to play an important role in Down syndrome pathology [29].

The miR-181 family is expressed in astrocytes and has been shown to be involved in their neuro-inflammatory response. Despite the current lack of information, MeCP2 has been found to be a target for miR-181 [30].

### MeCP2 in depression and cocaine abuse

One of the more fascinating aspects of chromatin’s epigenetic involvement is, in our opinion, the connection between chromatin alterations in response to environmental cues, such as early life stress (ELS), and the resulting behavioral output. It has now been fully demonstrated in rodents that maternal/parental care can affect gene expression [26, 28] and epigenetically affect the offspring in a trans-generational way [108]. More importantly, preclinical studies suggest that early life stressors, such as inconsistent and harsh parental discipline on their children [109], can result in increased stress responses leading to depressive disorders later on in adulthood [110]. At the molecular level, as will be discussed in the next paragraphs, an important part of the connection regards alterations in the function of the brain-derived neurothrophic factor (Bdnf) gene [111] that encodes for a member of the neurtrophin family of growth factors and its involvement in many important brain functions. It is a long gene with 4 promoters, which transcribe 4 mRNAs containing one of the four 5′ noncoding exons (I, II, III, or IV) spliced to the common 3′ coding exon [112], and is regulated by MeCP2. In addition to *Bdnf,* MeCP2 regulates the expression of many other similarly long genes that encode for proteins such as the calcium/calmodulin-dependent Camk2d kinase and those involved in axon guidance and synapsis formation [72]. Hence, as we have previously mentioned, is not surprising that MeCP2 in conjunction with epigenetic neuronal chromatin modifications are involved in many alterations of brain function that result in a plethora of neurological and psychiatric disorders [4, 113–120] (Fig. 2). The integration of environmental effects, such as stress, and the genetic and epigenetic modifications underlie what is currently known as synaptic and behavioral megaplasticity [121, 122]. In this section, I focus on two of them that have broad important social connotations in our current era: major depressive disorder (MDD), which has been quite extensively studied in recent years, and addiction (focusing on cocaine), for which a large number of studies are also available.

Whilst the genetic risk factors of both depression [123, 124] and addiction [125] are being established, the biochemical details and molecular mechanisms involved in the epigenetic counterpart are further ahead in their elucidation. At the chromatin level in a general mechanism, neuronal stimulation results in a Ca^2+^ influx that triggers the action of neuronal kinases (such as Camk2d) that phosphorylate different substrates, including MeCP2, CREB (cAMP response element-binding protein) and histone H3S10, amongst others [126]. Phosphorylation of MeCP2 weakens its interaction with chromatin, and phosphorylation of CREB allows it to bind to CBP (CREB-binding protein a histone acetyl transferase) [127], which acetylates histones and leads to a further chromatin relaxation, as was described in the previous section. All these modifications are conducive to a more open chromatin conformation, which enhances the accessibility of transcriptional co-activators to *cis* acting regulatory elements, like MEF2C, which results in gene activation (such as that of *Bdnf*) [126, 128].

One of the first molecular connections between ELS and depression was established through the arginine vasopressin gene (*Avp*). It was discovered that ELS was able to control the DNA methylation dynamics in post-meiotic neurons (see previous section), to result in stable persistent hypo-methylation of *Avp* expression that triggers the neuroendocrine and behavioral changes often observed in depression [129]. Early *Avp* derepression is driven by neuronal activity that results in the Ca2 + - Camk2d dependent MeCP2 phosphorylation and chromatin dissociation described in the previous paragraph, followed by DNA hypo-metylation. A vicious cycle is thus established in which MeCP2 occupancy uncouples from the original stimulus, leading to the ELS hard-coding at the level of DNA methylation [130]. The situation is by far more complex, and in addition to *Avp*, MeCP2 also regulates the ELS-dependent programming of other genes, such as *Crh* (corticotropin releasing hormone) and *Pomc* (Proopiomelanocortin) [131], all of which enhance the hypothalamic-pituitary-adrenal (HPA) axis which drives the ELS response, and are driven initially by MeCP2 S421 phosohorylation [48].

Whilst *Avp, Crh,* and *Pomc,* explain the connection between ELS and depression, *Bdnf*, as expected for any neuronal disturbance, also plays a very important role in depression [111], and its expression is decreased in stress and depression [132]. With the use of antidepressants, it has been shown that H3K27 methylation and histone deacetylation increase at the *Bdnf* III and IV promoters, a process that can be reverted with the use of histone methylation and HDAC5 inhibitors in mice [133]. It has been shown that the antidepressant citalopram decreases the levels of H3K27me3 at promoter IV of *Bdnf* in humans [134]. The histone PTM involvement in the stress-mediated neuronal response goes far beyond those briefly described here, and the reader is referred to [135] for a more comprehensive description.

MicroRNAs are also involved in depression. An increase was observed in the levels of miR-132 in the hippocampus of a rat model of stress-induced depression, and also in peripheral blood samples of patients with MDD. As expected from its role in MeCP2 homeostasis, a negative correlation between the expression levels of miR-132 and those of MeCP2 and BDNF was observed in these studies [11]. Also, high levels of miR-144-5p were detected in the plasma of MDD patients when compared to healthy controls, suggesting that miR-144-5p can be used as a biomarker for the disease [136].

Cocaine abuse and MDD share some similarities, let alone the fact that ELS is often a common risk factor for addiction [137, 138], which is considered a brain disorder of experience-dependent neuroplasticity [139]. Like ELS, cocaine affects the DNA methylation dynamics [140, 141] and in particular, within the nucleus acumbens (NAc) [142], a reward-related central region of the brain, both for 5mC and 5hmC [143]. Hence, it also affects MeCP2 [12, 144] and *bdnf* expression [145, 146].

The role of MeCP2 in cocaine addiction involves different aspects of the protein metabolism and its gene regulation and, in particular, the calcium-dependent MeCP2 S421 phosphorylation, one of its important PTMs that plays a crucial role [97, 147, 148]. The neuronal-activity mediated phosphorylation of MeCP2 S421 has been shown to have important transient and permanent effects in drug abuse and ELS respectively [129].

Of particular interest to this review is the interplay at the chromatin level between the induced levels of MeCP2 expression observed during cocaine intake [149] and the changes in histone acetylation [150]. After repeated (chronic) exposure to cocaine, the global levels of histone acetylation in the cocaine-targeted GABAergic neurons were observed to decrease in general agreement with the HDAC-mediated MeCP2 repressive activity [144]. However, the situation appears to be not that simple. In what could be considered a seminal paper on this topic, it was shown that an increase in the levels of histone H4 hyperacetylation in NAc occurs at the promoters of certain genes (such as the immediate early gene *c-Fos*), and is observed within 30 minutes of a single cocaine injection (acute exposure). However, the effect fades away during chronic exposure. Conversely, the levels of H3 acetylation increased at promoters of genes such as *Bdnf* and *Cdkl5* [151]. A genome-wide ChIP-chip analysis using antibodies against pan-acetylated H3/H4 has, interestingly, revealed that the increases in acetylation after chronic exposure do not affect the genome randomly [152], but rather increase the magnitude of their promoter distribution [150]. They have also confirmed the lack of overlap between H3 and H4 acetylated promoters [152]. All of this indicates that the cocaine-induced histone acetylation chromatin remodelling may be different for histones H3 and H4, in agreement with their different structural roles [153, 154].

Along with the shared molecular mechanisms with MDD, the cocaine-induced increase in MeCP2 represses the transcription of miR-132/miR-212 micro RNAs, which reduce the miRNA repression of *bdnf* through the feedback loop described in the previous section [139, 155]. Furthermore, it has been shown that in the dorsal striatum of rats, the expression of miR-212 is increased in those that show compulsive-like cocaine-taking behavior [156].

We would like to close this chapter with another intriguing aspect, which is that involving gender [157]. As was mentioned earlier, both depression and drug addiction share genetic and epigenetic contributions. It has now been well documented that women have a higher genetic predisposition to depression [158], which in turn may lead to a potential higher risk of drug abuse, which also exhibits important differences between both sexes [159]. Epigenetic studies of the brain have only recently started grasping this issue, but they have been postulated to also have a role in risk and resilience to mental health between the sexes [160]. For instance, 248 genes and loci associated with synaptic function were identified in mouse brains, with increased H3K4me3 in females [161]. Perinatal testosterone exposure resulted in important alterations of the DNA methylome [162], underscoring the contribution of the hormonal component [163]. Therefore, it is very important that when conducting future research in any of the areas described in this work, including those directly related to MeCP2, attention be paid to the sex differences and the differential involvement of gonadal hormones [164], the epigenetics new frontier [165].

## Conclusions

It has already been twenty-four years since the first description of MeCP2 [16, 17]. For a long time now we have been trying to decipher its mysteries. Significant progress has been made, yet many ‘heavy enigmas’ still remain. In this review, we have analysed the relevance and potential implications of this chromosomal protein, an abundant transcription regulator in neurons, for its architectural and functional role within the chromatin context. It is clear that in neuronal nuclei, MeCP2 is very weakly bound to chromatin, as a highly significant part of it is easily detached under very low ionic strength conditions, and it is bound to highly accessible nuclease domains [9]. We propose a model (Fig. 4) to account for these observations and their apparent disparity with the originally repressive role assigned to the protein. Whether the model is correct will require further experimental evidence. It will be critical at this point to be able to distinguish between the two MeCP2-E1 and MeCP2-E2 isoforms.

Finally, and although the broad attention by the scientific community to MeCP2 was triggered by the discovery of its massive involvement in RTT [2], a neurodevelopmental disease of autistic characteristics, its massive presence in the brain, and in neurons in particular, indicate its potential for a broader group of brain pathologies [4]. To underscore this, we have focused here on a brain disease (MDD) and on the neuronal disturbances resulting from cocaine abuse, both of which are highly pervasive issues within our society. A substantially large amount of information about some of the molecular details involved has been gathered for both of them. They provide an excellent example of how some of the critical epigenetic components that operate in the brain (DNA methylation, MeCP2 phosphorylation, histone acetylation and microRNAs) are intertwined. Similar mechanisms can be envisioned to participate in many other neurological disorders, and are currently being deciphered [4]. It will be important to take into consideration the relevant gender epigenetic differences [165].

## Abbreviations

*Avp*: arginine vasopressin gene
Bdnf: brain-derived neurotrophic factor
BER: base-excision repair
Camk2: Calcium/Calmodulin-Dependent Protein Kinase II
CBP: CREB-binding protein a histone acetyl transferase
Cdkl5: cyclin-dependent kinase-like 5
ChIP: chromatin immuno-precipitation
CREB: cAMP response element-binding protein
*Crh*: corticotropin releasing hormone gene
Dnmt: DNA methyl-transferases
ELS: early life stress
Fos: FBJ murine osteosarcoma
FRAP: fluorescence recovery after photobleaching
HDAC: histone de-acetylase
Hipk2: homeodomain-interacting protein kinase 2
Hipk2: homeodomain-interacting protein kinase 2
HMGN: high mobility group nucleosomal
HPA: hypothalamic-pituitary-adrenal
Igf2: insulin-like growth factor 2
MBD: methyl binding domain
MDD: major depressive disorder
MeCP2: methyl CpG binding protein 2
MEF2C: factor myocyte enhancer factor 2C
miRNA: microRNA
MRE: miRNA response elements
NAc: nucleus acumbens
NCoR: the nuclear receptor co-repressor
NCP: nucleosome core particle
Npas4: neuronal PAS domain pritein 4
NRL: nucleosome repeat length
PEST: enriched in proline, glutamic, serine and threonine
*Pomc*: Proopiomelanocortin gene
PTMs: post-translational modifications
RNF4: RING finger protein 4
RTT: Rett syndrome
Tet: ten eleven translocation
UPS: ubiquitin proteasome system
